# A Day Amongst the Surgeons of Philadelphia

**Published:** 1870-12

**Authors:** John E. Owens

**Affiliations:** Chicago


### ARTICLE V.—*A Day amongst the Surgeons of Philadelphia.* By JNO. E. OWENS, M.D., Chicago.

Through the kindness and courtesy of some of the most eminent surgeons of the Quaker City, I have been enabled to see very much that is interesting and profitable. I send you a few jottings of what has passed under my notice, hoping they may find a place amongst the valuable pages of the JOURNAL.

Prof. Pancoast gave me the particulars of a case of rhinoplasty, in which his son included, in the flap taken from the forehead, periosteum sufficient to produce new bone for the nose. I was sorry not to have seen this case, but it was impossible, as the patient had left the city. Prof. Pancoast's rule in this operation is to make the new nose one-third thicker and longer than the normal size. He applies, at the proper time, a gutta percha mould, with which, and a stuffing of cotton upon the inside of the new nose, he succeeds in giving this lump of flesh a shape. This mode of including the periosteum in the flap for the nose, originated in Berlin, with Langenbeck, who is a master in the art of plastic surgery, and who has taught us the full value that is attached to the preservation of the periosteum in bony resections. I believe, however, that, at present, Langenbeck does not look so favorably upon the periosteal transplantation method in rhinoplasty. Prof. Agnew, who has experimented considerably with periosteum, says that the new bone is deposited in nodules, and that even these do not always occur.

Professor Agnew was good enough to show me a pair of forceps which he had devised for the removal of large uterine polypi. The instrument is strong, and composed of a male and female blade, which may be introduced after the manner of the obstetrical forceps—one blade at a time. A lever of the third kind, attached to one handle, and passing through the second, is worked with the disengaged hand, after the instrument has been applied to the pedicle; and thus moving one toothed blade over the other, the tumor is chewed off. Of course there is no hæmorrhage. I saw him also use skin plaster or prepared peritoneum of the bullock for closing the eyelids after extraction of cataract. He has used this organic substance in the ligation of several large arteries, cutting off the ends of the organic ligature close to the vessel, and closing the wound over it. The result was satisfactory. This skin plaster may be ordered of Tiemann & Co.

Prof. Gross being absent in Baltimore, his son, Dr. S. W. Gross, conducted the college clinic. Delayed union of fractured radius was the first case presented. This was treated with the ivory pegs—Dieffenbach's method. Silicate of Potash was recommended as an immovable dressing in suitable cases, and used in this one. It is certainly an elegant substitute for plaster of Paris, or starch, and may be most conveniently applied with an ordinary paint brush. The second was a case of hydrocele, in which the following procedure was recommended: With an ordinary sharp-pointed bistoury, puncture the tumor in order to drain off the fluid, and, about a week subsequently, operate for the radical cure by injection. At the end of this time Dr. Gross teaches that the superficies of the vaginal tunic having very much decreased, a smaller surface is presented for inflammation at the time of the injection. Such a precaution may well be taken when the sack is very large, or when violent inflammation can be anticipated; but, in general, this preliminary is unnecessary, as the injection of strong Tr. Iodinii is sufficient. There is always an abundance of diseases of the urinary organs at the college. In the treatment of stricture of the urethra, rupture has become quite common; and the result of this method, so far as I have been able to learn, has been very gratifying. Richardson's, Voillemier's and Holt's dilators have been used for this purpose. A case of No. 4 stricture at the bulbous urethra was ruptured with Richardson's instrument, and immediately a No. 18 steel bougie was passed. The rents in the stricture are filled up by new tissue, and new mucous membrane is produced at the points of rupture. During the reparative process, of course dilatation by the passage of bougies is persevered in. A more lasting result is claimed for this than for any other method. In the next case of stricture, every justifiable effort was made to pass an instrument, but without success. The canal was irregular and tortuous; the urine dribbled away; there was hypertrophy and induration of the penis; œdema of the prepuce; the perineum was swelled, and deformed by scars; fistulous openings discharged urine, and here and there pus; the landmarks of the perineum were effaced. In such cases, tapping the membranous portion of the urethra is practiced. The knife is introduced not far from the verge of the anus, the only guide being the finger in the rectum. This relieves the parts, in front, from irritation, and frequently in a short time the improvement is such that an instrument may be passed.

I am very much indebted to Dr. Maury for the pains he took to show me two cases of ectropion vesicæ, upon which he had operated some time previous to my visit. Dr. Maury has immensely improved the condition of these patients in the following manner: The redundant folds of skin in the groin, covering hernial protrusions, and extending towards the perineum, were made available by carrying an incision across the perineum, making the extremities of the former look towards the tuberas ischiorum. This flap was marked out sufficiently large to oblige the operator to crowd it over and around the cleft in the abdomen. It was dissected up with its base at the lower margin of the cleft, and turned over the latter, like the leaf of a book, so that its cutaneous surface was presented to the ectropion. The upper edge of this flap, thus applied, was inserted beneath the integument above the cleft, and the edges were united by numerous points of suture. When the dissection of this large square flap reached the penis, the latter was liberated by a semi-lunar incision, and permitted to fall through the flap, and remain below it.

One other point. The penis, in these cases, is frequently placed so high that the urine very readily escapes over the sides of the cleft. In order to obviate this as much as possible, as it would certainly interfere more or less with the union of the flap, the doctor drew down the penis, and fixed it with a ligature. The cicatrical contraction had, to a great extent, forced back the hernial protrusions which, perhaps, always exist. In one case the cleft is perfectly closed. Of course there is no power of retaining urine, and the patients wear a rubber urinal with comfort. In the second case a small opening still exists, which can be very readily closed.

Dr. Maury will probably publish these cases, with illustrations, in "The Photographic Review." This is a new bi-monthly, illustrative of interesting cases, with notes. This novel addition to medical literature will prove welcome, as the superiority of photography to other means, for illustrating disease, has been very fully tested. The co-operation of the different gentlemen connected with the large hospitals has been secured, and material will be supplied in abundance. I have received the first issue (October, 1870), containing very excellent photographs of Multilocular Hydatid Tumor of the Leg, with notes by Prof. Gross; of Meningocele, with notes by Prof. Agnew; of Horny Tumors of the Face, with notes by W. H. Pancoast, M.D.; and of Keloid Tumors in the negro, under the care of F. F. Maury, M.D. At the end of the year the six numbers may be bound together, forming a handsome volume. This journal is well worth six dollars, the price of subscription, which may be sent to J. B. Lippincott & Co., Philadelphia.

Dr. Maury also had the kindness to present me with the report (reprinted from the "American Journal of the Medical Sciences," April, 1870,) of a case of stricture of the œsophagus, in which he performed gastrotomy. I copy the principal points from the report.

In May, 1868, the patient, aged 25, was suddenly seized with choking sensations and violent vomiting. Such paroxysms soon became of daily occurrence, and the saliva upon being swallowed shortly afterwards rose to the mouth. Deglutition at times was readily performed for several successive days. Again, for prolonged periods, not a morsel of food could be retained for more than a few moments. The diet consisted of broths, soups, and an occasional glass of wine. He began to emaciate and lose strength, and sought advice in July, 1868. Upon exploration with a gum-elastic bougie, somewhat below the medium size, a diminution in the calibre of the œsophagus was readily detected near its cardiac orifice; but at no subsequent period could even a small instrument be made to pass the stricture. No history of cancer in his family, and no irritating substance had been swallowed. There was a feeling of constriction and suffocation upon the injection of food. No pain at the seat of stricture. The patient acknowledged having had, at the age of seventeen years, an indurated chancre and buboes, which were followed by a papular eruption. The strength was sustained as much as possible, and hydrarg., bichloride, and potas. iod., were exhibited. After Jan. 1st, 1869, the tortures under which the patient had been laboring were aggravated; emaciation had become extreme; in the early part of April prostration confined him to his bed. He was as helpless as a child. Agonizing pains in the extremities, but more especially along the superficial surface of the tibiæ, now supervening, deprived him of rest both night and day. At the end of three weeks, he began to rally, and, after persevering efforts, to swallow small quantities of liquid. Having gained sufficient strength to leave his bed, he even went into the open air for short walks. About the middle of May he could no longer swallow anything. Resort was now had to enemata (brandy, beef extract, and milk punch,) upon which he subsisted for six weeks. The patient now earnestly importuned for an operation, but at this time, yielding to the earnest solicitations of the patient was considered unjustifiable. He continued to sink. The fast failing strength was sustained by enemata, and the pains controlled by morphia. Unsatisfied hunger caused the greatest distress. Finally, after consultation, it was decided to open the stomach. June 25th, 4 P. M. The patient, under the influence of chloroform, was placed upon his back, the left side being somewhat elevated by means of folded blankets. A curvilinear incision, the convexity presenting towards the median line, was commenced at the external extremity of the seventh intercostal space, and carried downwards and outwards for nearly four inches, exposing the sheath of the rectus muscle, which was slit upon a groved director. The fibres of the rectus were next separated, partly with the fingers, partly with the scalpel, the cutting edge of the latter being used as little as possible, with the view of avoiding hæmorrhage. The posterior lamella of the sheath of the rectus, the transversalis fascia and the peritoneum were next successively divided on a director, when the stomach was seen projecting below the margin of the liver. It (stomach) was firmly grasped by the forceps, and a curved needle, armed with a silver wire, was passed through its anterior wall, in the direction of the vertical line of the body, the points of entry and exit of the body being about an inch and a quarter apart. Two other needles, armed in the same manner, were then passed from left to right, and made to cross the first at right angles, the wires including that part of the stomach to be opened—a point about two inches to the left of the pylorus. The first wire was then withdrawn, and, by means of a pair of scissors, the stomach was opened in a perpendicular direction to the extent of an inch. The wires previously passed were thus exposed, and, by dividing them, the two sutures were converted into four. There was no escape of fluid into the peritoneal cavity. The viscus was now attached to the edges of the opening in the abdominal walls by numerous silver sutures. A tube, with a single flange, such as is employed when establishing gastric fistule in dogs, was inserted into the stomach, the flange resting upon the edges of the external wound and secured by tapes. The wound through the abdominal parietes was then closed by ordinary sutures. Two hours later the patient began to sink, and at six o'clock, the following morning, died. Post mortem nine hours after death. Body much emaciated; small intestines and stomach contracted and empty; peritoneum perfectly smooth and uninjected; all the internal viscera normal; no enlargement of lymphatic glands. A dense, firm stricture of the œsophagus was observed just within its cardiac orifice. The stricture produced such complete obliteration of the calibre of the œsophagus as scarcely to admit of the passage of a probe. A microscopical examination of its substance, made by Drs. Pepper and Tyson, gave no evidence as to its nature except that it probably was not cancerous.*

I need scarcely tell you that the faculties of the hospitals and colleges here have expressed themselves as adverse to conducting clinical instruction in the presence of students of both sexes. I cannot give you the views of the profession here, in a more condensed form, than is contained in the following "Remonstrance against clinical instruction being given to classes composed of both sexes," and signed by the faculties of the University of Pennsylvania, Jefferson Medical College, the Pennsylvania, Philadelphia, Episcopal, Wills, St. Joseph's, St. Mary's, the German, Children's, Charity, and Howard Hospitals; by the Northern and Philadelphia Dispensaries; and by the physicians at large of the city of Philadelphia:

"I. Clinical instruction in practical medicine demands an examination of all the organs and parts of the body, as far as practicable; hence, personal exposure becomes for this purpose often a matter of absolute necessity. It cannot be assumed, by any right-minded person, that male patients should be subjected to inspection before a class of females, although this inspection may, without impropriety, be submitted to before those of their own sex. A thorough investigation, as well as demonstration, in these cases—so necessary to render instruction complete and effective—is, by a mixed audience, precluded; while the clinical lecturer is restrained and embarrassed in his inquiries, and must, therefore, fall short in the conclusions which he may draw, and in the instruction which he communicates.

"II. In many operations upon male patients, exposure of the body is inevitable, and demonstrations must be made which are unfitted for the observation of students of the opposite sex. These expositions, when made under the eye of such a conjoined assemblage, are shocking to the sense of decency, and entail the risk of unmanning the surgeon—of distracting his mind, and endangering the life of his patient. Besides this, a large class of surgical diseases of the male is of so delicate a nature as altogether to forbid inspection by female students. Yet a complete understanding of this particular class of diseases is of pre-eminent importance to the community. Moreover, such affections can be thoroughly studied only in the clinics of the large cities, and the opportunity

* This is the first operation of gastrotomy for the obstruction of the œsophagus in this country, and the tenth on record. All the cases died within a few hours, except one, which lived twelve days.

for studying them, so far from being curtailed, should be extended to the utmost possible degree. To those who are familiar with such cases as are here alluded to, it is inconceivable that females should ever be called to their treatment.

"III. By the joint participation, on the part of male and female students, in the instruction and in the demonstrations which properly belong to the clinical lecture room, the barrier of respect is broken down, and that high estimation of womanly qualities, which should always be sustained and cherished, and which has its origin in domestic and social associations, is lost, by an inevitable and positive demoralization of the individuals concerned, thereby entailing most serious detriment to the morals of society."

